# Effect of *Scutellaria baicalensis* supplementation on sow milk yield and litter growth performance in Danish sows

**DOI:** 10.1093/tas/txag009

**Published:** 2026-01-23

**Authors:** Takele Feyera, Maria J Carrión-López, Delphine Gardan-Salmon, Alice Hamard, Fabrice Robert, Jan V Nørgaard

**Affiliations:** Department of Animal and Veterinary Sciences, Aarhus University AU, Viborg, Dk-8830, Denmark; CCPA Group, Janzé, 35150, France; Department of Animal and Veterinary Sciences, Aarhus University AU, Viborg, Dk-8830, Denmark; CCPA Group, Janzé, 35150, France; CCPA Group, Janzé, 35150, France; CCPA Group, Janzé, 35150, France; Department of Animal and Veterinary Sciences, Aarhus University AU, Viborg, Dk-8830, Denmark

**Keywords:** Lactation, milk yield, oxidative stress, litter performance, secondary metabolites

## Abstract

S*cutellaria baicalensis* root possesses potent anti-inflammatory and antioxidant properties, along with its ability to stimulate mammary cells, thereby enhancing milk yield. The present study investigated the effect of SB supplementation in lactation diet on colostrum and milk production, litter survival and growth, oxidative status, and inflammation in lactating sows. On day 108 of gestation, 24 sows were assigned to either a control group (**CON**; *n* = 12) that were fed according to the Danish nutrient standard or a CON diet supplemented with S*cutellaria baicalensis* root (**SB**; *n* = 12) and fed the diet until day 28 of lactation. Piglets were individually weighed at birth and weekly during lactation. Colostrum was sampled during farrowing, while milk was collected weekly during lactation. Blood samples were collected on day 112 of gestation, day of farrowing, day 3, 10, and 17 of lactation and analyzed for oxidative and inflammation biomarkers. Supplementation with SB did not affect litter number at birth but increased average birth weight of the piglet (*P* = 0.002). Litter weight was greater in SB supplemented sows (*P* < 0.05) during lactation. Colostrum concentration of lactose increased (*P* < 0.01) but that of protein and solid-not-fat (both *P* < 0.01) decreased with SB supplementation. Total daily protein secretion in milk increased (*P* = 0.02), while lactose (*P* = 0.09) and solid-not-fat (*P* = 0.07) tended to increase with SB supplementation. Supplementation with SB tended to increase milk yield (*P* = 0.06), litter number in lactation (*P* = 0.08), and litter survival (*P* = 0.10) compared to non-supplemented sows. Supplementation with SB affected neither oxidative stress nor inflammation status biomarkers. In conclusion, SB supplementation increased piglet birth weight, sow milk production, and litter growth performance during lactation, but did not influence oxidative status and inflammation.

## Teaser

The study indicated that *Scutellaria baicalensis* did not influence oxidative status, inflammation and farrowing outcomes but improved sow milk yield and litter performance during lactation.

## Introduction

Increased sow prolificacy has led to larger litter sizes, which negatively impact piglet birth weight, vitality, survival, and growth. During the final trimester of gestation, fetal development accelerates markedly, with approximately 35% of total fetal weight gained in the last 10 days before parturition (Wu et al. 1999; McPherson et al. 2004). This rapid growth substantially increases the metabolic demands on gestating sows. Conversely, sows often experience a reduced appetite during the early postpartum period (Ladirat 2024), which can make it difficult for them to consume adequate feed to meet the nutritional demands of milk production. As a result, the imbalance between nutrient requirements and intake during late gestation and early lactation may predispose the sows to mobilize body reserves to compensate for the deficit. This catabolic state can lead to overproduction of reactive oxygen species (**ROS**), contributing to maternal oxidative stress (Tan et al. 2015). Oxidative stress can lead to immunosuppression, increased stillbirth, induce inflammation, ultimately reduce feed intake and milk yield (Wang et al. 2022). The reduction in milk yield can negatively impact piglet growth and survival (Kaiser et al. 2018b), emphasizing the need to explore dietary strategies to mitigate oxidative stress in sows.

Dietary antioxidants of plant derived natural bioactive compounds have been shown to enhance sow health and litter growth (Reyes-Camacho et al. 2020; Parraguez et al. 2021). Recently, growing interest has been directed toward the use of bioactive plant extract as dietary supplements to mitigate oxidative stress and inflammation in late gestating and lactating sows (Li et al. 2022; Liangkai et al. 2022; Chen et al. 2023). These studies have reported that plant extracts exert beneficial effects by regulating nutrient metabolism and enhancing serum antioxidant status, thereby reducing oxidative stress in sows, which in turn improves litter growth performance. *Scutellaria baicalensis* (**SB**), a traditional Chinese medicine flowering plant, is well known for its anti-inflammatory, antioxidative, and hepatoprotective properties, primarily attributed to its bioactive flavonoids such as baicalin and baicalein (Yin et al. 2021; Liao et al. 2021; Jia et al. 2021; Wang et al. 2022). Several studies have demonstrated the anti-oxidative capacity of SB and its ability to increase milk yield in dairy cows (Olagaray et al. 2019; Perruchot et al. 2019), as well as its role in reducing apoptosis in bovine mammary epithelial cells via activation of the Nrf-2 signaling pathway (Perruchot et al. 2019). Moreover, improved growth performance and nutrient digestibility have been reported in finishing pigs supplemented with SB (Liu et al. 2016). However, evidence on the effects of SB supplementation on oxidative status, as well as on productive and reproductive performance of hyperprolific modern sows, such as Danish sows, remains limited.

It is hypothesized that SB supplementation during the peripartum and lactation periods may help reduce oxidative stress and inflammation in sows, enhance mammary gland secretory function, and ultimately improve milk production and litter growth. Therefore, this study aimed to investigate the effects of SB supplementation during the transition and lactation periods on oxidative stress and inflammatory biomarkers, milk production and the subsequent impact on litter survival and growth in Danish sows.

## Materials and methods

The animal experiment procedures and care of animals under study were carried out in accordance with the Ministry of Food, Agriculture and Fisheries, The Danish Veterinary and Food administration under act 474 of 15 May 2014 and executive order 2029 of 14 December 2020. Rearing, housing, and sampling were in coherence with Danish laws for the care and use of animals for research purposes. The Danish Animal Experimentation Inspectorate approved the study protocol and supervised the experiment with the license number 2023-15-0201-01594.

### Handling of sows and piglets

The experiment included 24 DanBred Landrace × DanBred Yorkshire sows of 2–4 parties and 12 sows per dietary treatment. The number of sows allocated to each dietary treatment was based on the research facility’s capacity to manage lactating sows in crate housing. A formal power analysis was not performed to determine the sample size, as the primary aim of the study was not to evaluate dietary effects on production performance. Instead, the focus was on assessing the impact of diets on oxidative and inflammatory status during the transition and lactation periods through repeated sampling. On day 107 of gestation, sows were stratified for body weight and backfat thickness and randomly assigned to one of two dietary treatments from day 108 of gestation until weaning on day 28 of lactation. Sows were individually housed with their litter in non-bedded farrowing crates (2.7 × 1.7 m) that featured partly slatted floor throughout the experimental period. The room temperature was kept at 20°C, and the light was turned on from 0630 to 1830 h and from 2230 to 0030 h during the night meal. Around the time of farrowing, the light was turned 24 h a day. Each farrowing crate had a separate creep area for piglets, including cover, floor heat, and an infrared heating lamp to keep the ambient temperature of the creep area around 32°C during farrowing. The temperature in the creep area was down regulated if the piglets were observed moving away from the creep area and turned off after the second week of lactation. All sows farrowed naturally without farrowing induction and farrowing was not physically monitored, thus farrowing assistance was not provided in this study. However, farrowing was observed from video surveillance to detect the onset of farrowing for the purpose of collecting blood and colostrum samples at the onset of farrowing. The number of total born, live born, and stillborn piglets were recorded after the end of farrowing. All live born piglets were ear-tagged, and their individual body weight was recorded, which was considered as their birth weight.

Litters numbers were standardized to have 14 piglets in each litter within 24 to 36 h after the onset of farrowing, except in one litter that was adjusted to 13 piglets to match with the number of functional teats of the sows, and all piglets were weaned on day 28 of lactation. Standard piglet management procedures such as teeth clipping, tail docking, iron injection, and castration were carried out according to the normal procedures of the pig herd at Aarhus University, research center Foulum, AU Viborg. During lactation, piglets were weighed individually once weekly on day 2, 7, 14, 21, and 28 of lactation to record litter weight gain, which in turn was used to predict sows’ milk yield. Piglets had no access to creep feed during the lactation period. Rectal temperature of the sows was measured on the day of farrowing, day 1, 2, and 3 of lactation. Body weight and backfat thickness of the sows were measured on day 107 of gestation, on day 2 and 28 of lactation. Backfat thickness was measured at 66 mm from the midline at the last rib on the right side of the sows using a Renco Lean-Meater^®^ digital backfat indictor (Renco Corp., Minneapolis, USA).

### Diet and feeding

During the period from day 108 of gestation until weaning on day 28 of lactation, sows were provided with a lactation diet based on wheat, barley, and soybean meal, which was formulated according to the Danish recommendation (Tybirk et al. 2018). Table 1 shows the dietary ingredients as well calculated and analyzed chemical compositions of the diets. Sows received either a standard lactation diet formulated according to the Danish ­recommendation for lactating sows (**CON;** *n* = 12), or a CON diet supplemented with 0.2% of FEEDSTIM Sow (**SB**-CCPA Group Janzé, France; *n* = 12), providing 135 mg/kg of *Scutellaria baicalensis* via whole ground root with calcium carbonate as carrier. The root of *Scutellaria baicalensis* is a rich source of flavones, comprising approximately 90% baicalin, 8% baicalein, and 2% wogonin of the total flavone content (Zhao et al. 2016). The *Scutellaria baicalensis* premix was incorporated into the standard lactation diet formulation, with adjustments made to maintain equivalent sodium levels. FEEDSTIM Sow was included at the expense of limestone. This substitution is not expected to alter the overall calcium content of the treatment diet, as the carrier in FEEDSTIM Sow is calcium carbonate. Feed allowance was adjusted to 3.7 kg/d during the last week of gestation and gradually increased during lactation until it reached the maximum allowance of 8.8 kg/d on day 17 of lactation and maintained this feeding level in the remaining days of lactation until weaning. Feed allowance was further adjusted during lactation based on the number of suckling piglets registered on day 7, 14 and 21 of lactation at each weekly weighing. Specifically, the allowance was reduced by 5% for each piglet lower than 14, down to a minimum of 9 piglets per litter. The sows were fed three meals per day at 0700, 1500, and 2300 h using a Spotmix feeding system (SpotMix Schauer Agrotronic GmbH, Prambachkirchen, Austria). To calculate the realized feed intake, leftover feed was collected daily between 0900 and 1000. Once a week, feed samples were collected and stored at −20°C until analysis.

**Table 1:** Dietary ingredients, calculated and analyzed chemical compositions of the experimental diet.

Ingredients, %	CON	SB
** Wheat**	42.559	42.543
** Barley**	35.000	35.000
** Soybean meal**	15.822	15.825
** Sugar beet pulp**	2.000	2.000
** Vegetable oil, fat and soy**	1.134	1.138
** Limestone (granulated)**	1.385	1.194
** Monocalcium phosphate**	0.870	0.870
** Sodium chloride**	0.541	0.541
** L-Lys HCL (98.5%)**	0.293	0.293
** Premix^a^**	0.206	0.206
** L-Thr (98.5%)**	0.107	0.107
** DL-Met**	0.065	0.065
** E-vitamine**	0.018	0.018
** SB supplementation^b^**	…	0.200
**Calculated composition, %**		
** Dry matter**	87.62	87.62
** Starch**	44.30	44.30
** Crude protein**	14.97	14.97
** Ash**	5.40	5.40
** Crude fiber**	3.80	3.80
** Soluble dietary fiber**	4.40	4.40
** Insoluble dietary fiber**	12.70	12.70
** Fat**	3.20	3.20
**Analyzed, %**		
** Dry matter, g/kg feed**	87.50	87.50
** Crude protein**	14.60	14.80
** Ash**	4.70	4.80
** Fat**	3.50	3.50

a Supplied per kilogram of diet: 8960 IU retinol; 2000 IU 25-hydroxy vitamin D3 (Hy-D, DSM Nutritional Products, Basel, Switzerland); 167 mg α-tocopherol; 2.27 mg thiamin; 5.62 mg riboflavin; 22.7 mg niacin; 167 mg pantothenic acid; 3.35 mg pyridoxine; 5.94 mg folic acid; 0.02 mg cobalamin; 0.43 mg biotin; 4.32 mg menadion; 88.99 mg iron (FeSo_4_); 13.0 mg copper (CuSO_4_); 45 mg manganese (MnO), 103 mg zinc (ZnO); 0.23 mg iodine (Ca(IO_3_)_2_), 0.38 mg selenium (Na_2_SeO_3_).

b 0.2% of FEEDSTIM Sow (SB—CCPA Group Janzé, France), a Scutellaria baicalensis root with a calcium carbonate carrier providing, 135 mg/kg of Scutellaria baicalensis. The inclusion of 0.2% of FEEDSTIM Sow was made at the expense of limestone. However, this substitution is not expected to alter overall calcium content of the treatment diet, as the carrier FEEDSTIM Sow is calcium carbonate.

### Colostrum and milk sampling, and estimation of milk yield

A single colostrum sample was collected within an hour of the birth of the first piglet in the litter. Milk samples were collected on day 3, 10, 17, and 24 of lactation. Milk let-down was induced by injecting 0.20 mL oxytocin (10 IU/mL; Lovens Kemiske Fabrik, Ballerup, Denmark) into an ear vein. During established lactation, as many teats as possible on both sides were milked. However, during farrowing, only teats from either the left or the right side were milked, as one side is not accessible while the sows are farrowing. At each sampling time, 50 mL of colostrum and milk sample was hand-milked, filtered through gauze and stored at −20°C until analysis. Weekly milk yield of the sow was determined retrospectively based on weekly litter gain and litter number within each week using the Hansen et al. (2012) prediction model.

### Plasma sampling

A 9 mL blood sample was collected on day 112 of gestation, on day of farrowing, on day 3, 10, and 17 of lactation. Except on the day of farrowing, the blood samples were collected 4 h after the morning meal by puncturing the jugular vein. On the day of farrowing, the sample was collected by puncturing ear vein, and the blood sample was collected following birth of the first piglets in the litter. The blood sample was collected into a 10 mL heparinized vacutainer tube (Greiner Bio-One GmbH, Kremsmünster, Austria). The tube was immediately placed on ice until centrifugation. The blood samples were centrifuged at 1558 × *g* for 10 min at 4°C. The resulting plasma aliquots were then harvested into 1.5 mL tubes and stored at −20°C until further analysis.

### Analytical procedures

Chemical analyses on the diets were performed in duplicate. Dry matter content was determined by drying the sample at 103°C for 20 h in a forced air oven until a constant weight was achieved. Analyses of crude ash, nitrogen, and crude fat (EC 152/2009) followed the guidelines established by the Official Journal of the European-Commission (2009). Milk samples underwent analyses for fat, protein, lactose, solid-not-fat, and dry matter concentrations in triplicate using infrared spectroscopy (Milkoscan 4000, FOSS, Hillerød, Denmark). Total daily secretion of milk components was calculated as the product of milk yield and concentrations of the respective milk components.

Plasma alanine aminotransferase (**ALT**), aspartate aminotransferase (**AST**), gamma-glutamyl transferase (**GGT**), creatine kinase, glucose, lactate, triglycerides, and urea concentrations were determined according to standard procedures (Siemens Diagnostics® Clinical Methods for ADVIA 1650). Plasma haptoglobin was determined by a chemical reaction between haptoglobin and haemoglobin (PHASE^TM^ RANGE, Haptoglobin Assay, Tridelta Development Limited, Maynooth Co. Kildare, Ireland). Plasma concentration of non-esterified fatty acids (**NEFA**) was determined using the Wako NEFA C ASA-ACOD assay method (Wako Chemicals GmbH, Neuss, Germany). All analyses were performed using an autoanalyzer, ADVIA 1800® Chemistry System (Siemens Medical Solutions, Tarrytown, NY 10591, USA).

Superoxide dismutase (**SOD**) and catalase activity were determined by colorimetric assay (Arbor Assays, Michigan 48108 USA). Glutathione peroxidase (**GP**x) and malondialdehyde were analysed using ELISA assays (BioSite, EKX-4OYT15 and EKX-HSLYQ5). Total antioxidant capacity (**TAC**) was determined by colometry (Cell Biolabs, STA-360; San Diego, California, USA). The instructions given by the manufacturer were followed for all these analyses.

### Statistical analysis

The experiment was regarded as a complete randomized design in which sows were stratified for body weight and backfat thickness and assigned randomly to one of two dietary treatments. The statistical analyses were performed using the SAS procedure (version 9.3, SAS Institute Inc., Cary, NC). Parameters related to sow and litter performance, and colostrum compositions were analyzed using the MIXED procedure of SAS including treatment (CON, SB), parity (2, 3, 4) and treatment × parity as fixed effects. The incidence of stillbirth was analyzed using the GLIMMIX (assuming binomial distribution) procedure of SAS including treatment, parity, and treatment × parity as fixed effects.

Milk compositions were analyzed using the MIXED procedure of SAS including treatment, parity, days in milk (3, 10, 17, 24), and treatment × parity as fixed effects. Concentrations of malondialdehyde, antioxidants, haptoglobin, liver markers, and energy metabolites in the plasma were analyzed using the MIXED procedure of SAS including the fixed effects of treatment, parity, days relative to farrowing (−3, 0, 3, 10, 17), and treatment × parity. Feed intake and milk yield in lactation and litter performance during the lactation period were analyzed using the MIXED procedure of SAS including the fixed effects of treatment, parity, week of lactation (1, 2, 3, 4), and treatment × parity. For the repeated measurements in the above models, sow was included in the model as a random effect to account for repeated measurements by using variance component as the covariance structure. The data are reported as LSMEANS ± SEM, statistical significance was declared at *P* < 0.05, and a tendency was indicated at *P* *≤* 0.10.

## Results

### Performance

**Figure 1:**
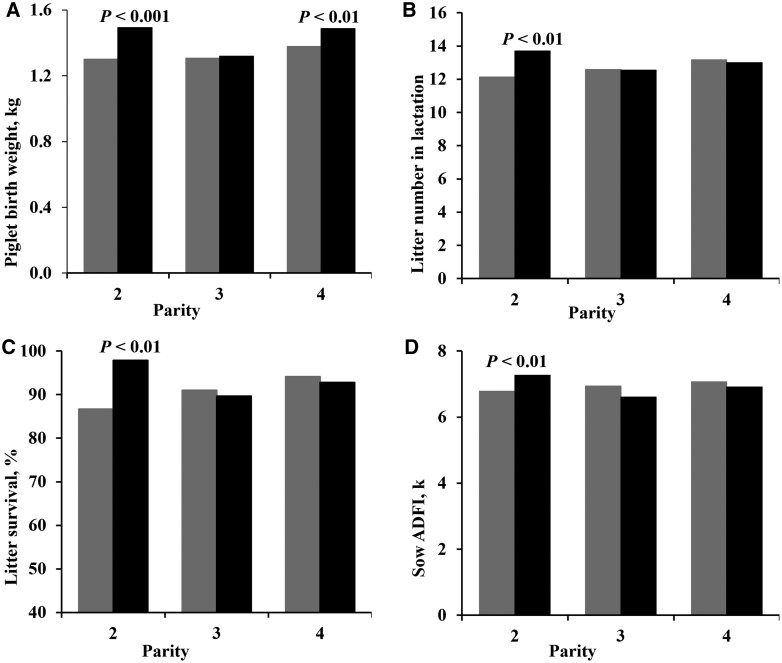
Interaction between dietary treatment and parity for a) piglet birth weight, b) litter size in lactation, c) litter weight in lactation, and d) average daily feed intake (ADFI) in sows fed either a standard lactation diet (gray bar) or a standard lactation diet supplemented with *Scutellaria baicalensis* plant (black bar) form day 108 of gestation until weaning on day 28 of lactation. *Scutellaria baicalensis* plant (SB) is blended in a calcium carbonate vehicle providing 135 mg/kg of Scutellaria baicalensis.

An interaction between dietary treatment and parity was observed for average piglet birth weight, in which second (*P* < 0.001) and fourth (*P* < 0.01) parity sows supplemented with SB gave birth to heavier piglets compared to the CON sows (Fig. 1a). Additionally, interactions between dietary treatment and parity were observed for litter number (Fig. 1b), litter survival (Fig. 1c), and sow average daily feed intake (Fig. 1d) in lactation. Accordingly, second parity sows supplemented with SB showed higher litter number, litter survival, and sow average daily feed intake compared to the CON sows (all *P* < 0.01).

Sows supplemented with SB gave birth to heavier piglets compared to piglets from the CON sows (*P* = 0.002; Table 2). Supplementation with SB tended to increase sow body weight loss during lactation (*P* = 0.06). Third parity sows gave birth to lighter piglets (*P* < 0.001) and weaned lower proportion of piglets weighing < 7 kg (*P* < 0.01) but had greater stillbirth rate (*P* < 0.01) compared to 2^nd^ and 4^th^ parity sows. The proportion of lighter piglets at weaning was numerically greater in sows fed the CON diet compared to piglets from sows fed SB supplemented diet.

**Table 2:** Productive and reproductive performances in sows fed either a standard lactation diet (CON) or a standard lactation diet supplemented with *Scutellaria baicalensis* plant (SB) from day 108 of gestation until weaning on day 28 of lactation.

Item	Diet			Parity				*P*-value		
	CON	**SB** ^1^	SEM	2	3	4	SEM	Diet	Parity	Diet*parity
**Gestation length**	116	116	0.3	116	116	116	0.4	0.84	0.67	0.16
**Sow BW at day 107 of gestation, kg**	271	272	5.7	267	268	280	7.8	0.94	0.38	0.99
**Sow BW at day 2 of lactation, kg**	255	259	5.4	253	253	266	7.5	0.54	0.37	0.85
**Sow BW change 2 to 28 of lactation, kg**	10.9	18.2	2.66	13.9	15.8	13.8	3.68	0.06	0.87	0.67
**Sow backfat at day 107 of gestation, mm**	11.6	10.8	0.62	12.5	10.9	10.2	0.86	0.35	0.13	0.95
**Sow backfat day 2 of lactation, mm**	11.1	10.3	0.54	12.1^a^	10.6^ab^	9.33^b^	0.74	0.23	<0.05	0.78
**Sow backfat change 2 to 28 of lactation, mm**	1.59	1.56	0.16	2.00^a^	1.40^b^	1.33^b^	0.22	0.99	<0.05	0.23
**ADFI in the last week of gestation, kg**	3.70	3.70	0.01	3.72	3.71	3.69	0.01	0.81	0.18	0.76
**Average piglet birth weight, kg**	1.16	1.26	0.02	1.26^a^	1.12^b^	1.26^a^	0.03	0.002	<0.001	0.02
**Litter birth weight, kg**	26.6	28.3	1.06	25.5	26.9	29.9	1.46	0.33	0.09	0.67
**Litter weight at day 2 of lactation, kg**	20.7	22.4	0.66	22.1	20.4	22.1	0.92	0.13	0.22	0.18
**Litter weight at weaning, kg**	104	111	4.1	106	107	109	5.6	0.22	0.92	0.56
**Number of live born piglets**	20.9	21.0	0.87	21.0	19.8	22.0	1.20	0.78	0.35	0.33
**Number of total born piglets**	22.1	23.5	1.16	21.5	22.9	24.0	1.60	0.57	0.50	0.42
**Stillbirth, %**	4.971	8.01	[2.42, 13.5]	2.33^b^	13.0^a^	7.31^b^	[0.82, 18.6]	0.20	<0.01	0.74
**Proportion of piglets <7 kg at weaning, %**	14.2	11.0	2.49	14.8^a^	6.34^b^	16.7^a^	3.44	0.42	<0.05	0.15
**Proportion of Piglet <8 kg at weaning, %**	33.0	23.8	4.83	31.1	29.9	24.2	6.69	0.17	0.72	0.36

1 Scutellaria baicalensis root extract with a calcium carbonate carrier providing 135 mg/kg of Scutellaria baicalensis.^a,b^Means within a row with different superscript differ (*P* < 0.05).

Litter weight in lactation was greater in sows supplemented with SB compared with litter weight from the CON sows (*P* < 0.05; Table 3). Supplementation with SB tended to increase milk yield (*P* = 0.06), litter size in lactation (*P* = 0.08), and litter survival (*P* = 0.10) compared to the CON sows. Milk yield, litter weight gain, and piglet gain increased (*P* < 0.001), while litter number and litter survival decreased (*P* < 0.01) with the progress of lactation.

**Table 3:** Rectal temperature and milk yield of the sows, and litter performances in sows fed either a standard lactation diet (CON) or a standard lactation diet supplemented with *Scutellaria baicalensis* plant (SB) from day 108 of gestation until weaning on day 28 of lactation.

Item	Diet			Parity				Week in Lactation (Week)					*P*-value			
	CON	**SB** ^1^	SEM	2	3	4	SEM	1	2	3	4	SEM	Diet	Parity	Week	Diet*parity
**Sow rectal temperature, °C^2^**	38.6	38.6	0.06	38.7	38.6	38.5	0.08	38.6	38.6	38.7	38.6	0.08	0.50	0.16	0.47	0.87
**Sow milk yield, kg**	12.5	13.2	0.33	12.6	12.9	13.1	0.45	8.71^c^	13.3^b^	15^a^	14.4^a^	0.46	0.06	0.67	<0.001	0.18
**ADFI during lactation, kg**	6.93	6.93	0.08	7.02	6.78	6.99	0.09	4.88^d^	6.94^c^	7.89^b^	8.01^a^	0.11	0.85	0.10	<0.001	<0.01
**Litter number in lactation**	12.6	13.1	0.18	12.9	12.6	13.1	0.26	13.7^a^	12.9^b^	12.5^b^	12.4^b^	0.26	0.08	0.25	<0.01	0.01
**Litter survival in lactation, %**	90.6	93.4	1.24	92.3	90.3	93.5	1.72	97.7^a^	92.1^b^	89.4^b^	88.9^b^	1.73	0.10	0.32	<0.01	<0.01
**Litter weight in lactation, kg^3^**	69.6	74.1	1.52	72.6	70.6	72.4	2.11	36.3^d^	59.8^c^	84.0^b^	107^a^	2.12	<0.05	0.68	<0.001	0.11
**Weekly litter gain, kg**	20.9	22.1	0.65	21.0	21.7	21.8	0.89	14.8^b^	23.5^a^	24.2^a^	23.4^a^	0.90	0.11	0.76	<0.001	0.27
**Piglet daily gain, g**	271	271	6.94	268	276	269	9.60	214^c^	279^b^	313^a^	278^b^	9.65	0.86	0.76	<0.001	0.19
**Intra-litter weight CV, %**	18.0	17.5	0.96	15.5^b^	17.4a^b^	20.4^a^	1.33	19.6	19.7	15.9	16.0	1.34	0.50	<0.05	0.06	0.32

1 Scutellaria baicalensis (SB) blended in a calcium carbonate vehicle providing 135 mg/kg of Scutellaria baicalensis.

2 Rectal temperatures of the sow were measured at day 0, 1, 2, and 3 of lactation, where day 0 is the day of farrowing.

3 Average weekly litter weight in lactation.

a,b Means within a row with different superscript differ (*P* < 0.05).

### Colostrum and milk compositions

Colostrum and milk composition responded distinctly to SB supplementation (Table 4). In sows supplemented with SB, colostral protein and solid-not-fat concentrations were lower (both *P* < 0.01), while lactose concentration was greater (*P* < 0.01) compared to CON sows. Similarly, milk from SB-supplemented sows had lower concentrations of fat (*P* < 0.01) and dry matter (*P* < 0.05) than that of CON sows. The amount of protein secretion in milk increased (*P* = 0.02), while that of lactose (*P* = 0.09) and solid-not-fat (*P* = 0.07) tended to increase with SB supplementation. Second parity sows had greater milk fat (*P* < 0.01) and dry matter (*P* < 0.05) concentrations than sows with parity three and four. Lactose concentration in milk increased (*P* < 0.001), while protein concentration decreased (*P* < 0.001) with the progress of lactation.

**Table 4:** Colostrum and milk concentrations in sows fed either a standard lactation diet (CON) or a lactation diet supplemented with *Scutellaria baicalensis* plant (SB) from day 108 of gestation until weaning on day 28 of lactation.

Item	Diet			Parity				Days in milk (DIM)					*P*-value			
	CON	**SB** ^1^	SEM	2	3	4	SEM	3	10	17	24	SEM	Diet	Parity	DIM	Diet*parity
**Milk, %**																
**Fat**	7.14	6.63	0.14	7.39^a^	6.65^b^	6.62^b^	0.21	6.76	7.28	6.79	6.70	0.18	<0.01	<0.001	0.11	0.82
**Protein**	5.00	5.06	0.05	4.98	5.02	5.09	0.07	5.49^a^	4.74^c^	4.84^c^	5.04^b^	0.06	0.22	0.56	<0.001	0.89
**Lactose**	5.08	5.09	0.02	5.06	5.12	5.08	0.03	4.98^c^	5.07^b^	5.15^a^	5.13^ab^	0.03	0.80	0.11	<0.001	0.46
**Solid-not-fat**	11.3	11.3	0.05	11.3	11.3	11.4	0.07	11.4^a^	11.2^b^	11.3^a^	11.4^a^	0.06	0.82	0.90	<0.05	0.84
**Dry matter**	18.1	17.6	0.14	18.4^a^	17.5^b^	17.8^b^	0.21	18.0	18.1	17.7	17.7	0.19	<0.05	<0.001	0.32	0.96
**Amount secreted in milk, g/day**																
**Fat**	874	884	30.9	909	877	850	44.3	625^b^	926^a^	1012^a^	952^a^	40.2	0.86	0.57	<0.001	0.97
**Protein**	622	665	17.7	613	646	672	25.4	501^c^	630^b^	727^a^	715^a^	20.1	0.02	0.23	<0.001	0.26
**Lactose**	636	672	19.0	634	665	663	27.2	431^b^	676^a^	774^a^	734^a^	24.7	0.09	0.53	<0.001	0.27
**Solid-not-fat**	1417	1494	40.5	1407	1468	1491	57.9	1015^c^	1486^b^	1705^a^	1616^ab^	52.6	0.07	0.51	<0.001	0.27
**Dry matter**	2236	2326	64.2	2264	2290	2289	91.9	1609^c^	2356^b^	2651^a^	2508^ab^	88.5	0.21	0.96	<0.001	0.63
**Colostrum, %^2^**																
**Fat**	4.65	5.19	0.32	4.78	4.83	5.14	0.45	…	…	…	…	…	0.20	0.77	…	0.27
**Protein**	20.4	18.2	0.55	20.0	18.5	19.4	0.78	…	…	…	…	…	<0.01	0.22	…	0.71
**Lactose**	3.40	3.56	0.05	3.43	3.52	3.49	0.07	…	…	…	…	…	<0.05	0.54	…	0.56
**Solid-not-fat**	23.2	21.4	0.48	22.8	21.6	22.4	0.67	…	…	…	…	…	<0.01	0.26	…	0.63
**Dry matter**	29.6	28.0	0.68	29.3	27.9	29.1	0.96	…	…	…	…	…	0.09	0.36	…	0.30

1 Scutellaria baicalensis (SB) blended in a calcium carbonate vehicle providing 135 mg/kg of Scutellaria baicalensis.

2 Colostrum samples were collected only at the onset of farrowing.

a–c Means within a row with different superscript differ (*P* < 0.05).

### Plasma metabolites

Apart from minor numerical differences, SB supplementation did not significantly affect markers of oxidative status, systemic inflammation, or liver health in sows (Table 5). Plasma concentrations of biomarkers related to oxidative status, systemic inflammation, and liver health status fluctuated throughout the transition and lactation periods. Notably, plasma malondialdehyde concentration was lowest on the day of farrowing (28.8 ng/mL), highest on day 3 of lactation (46.9 ng/mL), and intermediate (35.8–39.9 ng/mL) in the remaining days (*P* < 0.01). Catalase activity was lower on day 112 of gestation (*P* < 0.01), while concentration of GPx was greatest on day 17 of lactation (*P* < 0.01) compared to the remaining days.

**Table 5:** Plasma concentrations of oxidants, antioxidants, liver biomarkers, and energy metabolites in sows fed either a standard lactation diet (CON) or a standard lactation diet supplemented with *Scutellaria baicalensis* plant (SB) from day 108 of gestation until weaning on day 28 of lactation.

Item	Diet			Parity				Days in milk (DIM)						*P*-value			
	CON	**SB** ^1^	SEM	2	3	4	SEM	−3	0	3	10	17	SEM	Diet	Parity	DIM	DIM*parity
**Malondialdehyde, ng/mL**	39.6	37.3	3.47	39.6	39.0	36.6	4.86	39.6^b^	28.7^c^	46.8^a^	41.1^b^	35.7^b^	3.42	0.64	0.89	<0.001	0.07
**GPx, ng/mL^2^**	60.1	55.6	2.35	61.9	54.9	54.9	3.30	55.6^b^	55.7^b^	58.4^b^	53.5^b^	66.2^a^	2.83	0.18	0.20	<0.01	0.45
**Catalase, U/mL**	40.6	41.4	2.85	38.5	38.9	45.5	4.00	29.9^b^	44.5^a^	43.2^a^	44.3^a^	43.0^a^	3.09	0.85	0.35	<0.001	0.87
**SOD, U/mL^3^**	0.47	0.51	0.05	0.49	0.54	0.43	0.07	0.52^b^	0.81^a^	0.52^b^	0.32^^c^^	0.26^c^	0.05	0.60	0.51	<0.001	<0.01
**TAC, μM/L^4^**	325	325	11.1	327	320	328	15.6	343	296	324	326	336	14.6	0.98	0.89	0.16	0.80
**Haptoglobin, mg/mL**	1.88	2.00	0.15	2.15	2.11	1.54	0.21	1.74^b^	1.64^b^	2.69^a^	1.90^b^	1.70^b^	0.13	0.56	0.06	<0.001	<0.05
**ALA, U/L^5^**	49.7	51.0	2.34	50.9	50.4	49.8	3.28	47.7^b^	47.0^b^	52.4^a^	52.0^a^	52.7^a^	1.98	0.68	0.97	<0.001	0.68
**ASA, U/L^6^**	47.9	50.4	2.69	53.1	48.3	46.1	3.77	37.7^c^	47.2^b^	58.7^b^	48.1^b^	54.2^a^	2.75	0.52	0.35	<0.001	0.58
**GGT, U/L^7^**	24.3	24.6	1.76	24.8	23.5	25.1	2.46	24.9^b^	18.7^c^	23.8^b^	24.7^b^	30.2^a^	1.67	0.92	0.84	<0.001	0.14
**Creatine kinase, U/L**	1162	1392	115	1452	1288	1090	161	1062^b^	1317^b^	1735^a^	1107^b^	1161^b^	157	0.17	0.25	<0.01	0.23
**Glucose, mM/L**	5.56	5.41	0.08	5.70	5.45	5.31	0.11	4.70^b^	5.86^a^	5.75^ab^	5.56^b^	5.57^b^	0.10	0.20	<0.05	<0.001	0.95
**Lactate, mM/L**	2.26	2.06	0.14	2.02	2.05	2.41	0.20	2.67^a^	1.77^c^	2.14^b^	2.00^b^	2.21^b^	0.16	0.31	0.28	<0.001	<0.05
**Triglycerides, mM/L**	0.20	0.22	0.01	0.22	0.21	0.20	0.02	0.36^a^	0.21^b^	0.18^bc^	0.15^c^	0.15^c^	0.01	0.39	0.64	<0.001	0.62
**NEFA, μM/L^8^**	242	250	19.0	234	257	248	27.2	119^c^	465^a^	164^c^	316^b^	171^c^	30.0	0.77	0.77	<0.001	0.82
**Urea, mM/L**	3.84	3.78	0.11	3.77	3.99	3.67	0.15	3.72^b^	2.96^c^	3.70^^b^^	4.30^a^	4.36^a^	0.14	0.74	0.21	<0.001	0.55

1 Scutellaria baicalensis (SB) blended in a calcium carbonate vehicle providing 135 mg/kg of Scutellaria baicalensis.

2 Glutathione peroxidase.

3 Superoxide dismutase.

4 Total antioxidant capacity.

5 Alanine aminotransferase.

6 Aspartate aminotransferase.

7 Gamma-Glutamyl transferase.

8 Non-esterified fatty acid.

a–c Means within a row with different superscript differ (*P* < 0.05).

## Discussion

Due to the ban on in-feed antibiotics use in Europe, there has been a growing scientific interest in bioactive plant extracts as dietary alternatives to mitigate health-related risks in animal production. *Scutellaria baicalensis* root, rich in flavonoids like baicalin and baicalein, is known for its anti-inflammatory, antioxidative, and hepatoprotective effects. This study tested the hypothesis that SB supplementation during transition and lactation could reduce oxidative stress and inflammation in sows, thereby enhancing milk production and litter performance.

### Performance

The dietary intervention in this study showed no significant impact on the number of live born and total born piglets. The dietary intervention began during the final week of gestation, which was likely too late to significantly influence the number of total born piglets. Investigating the impact of SB supplementation on the total number of piglets born was beyond the scope of this study. Nevertheless, future research should investigate the supplementation of SB from early gestation to determine its potential influence on total born. A previous study that administered a mixture (1 g/kg) comprised of 50% SB, 30% *Lonicera japonica*, and 20% carrier product (wheat bran) from day 85 of gestation until weaning reported a greater number of live born piglets compared to non-supplemented sows (Wang et al. 2022), which is contrasting with the present finding. The results of Wang et al. (2022) may indicate that blends of botanical bioactive components exert synergistic effects or that their impact become more pronounced when provided over a longer period. In the present study, the average birth weight of piglets born from sows supplemented with SB was greater compared to those born from the CON sows. It was shown that 35% of fetal weight gain occurs during the final 10 days of gestation (Wu et al. 1999; McPherson et al. 2004). Cell proliferation and angiogenesis are critical process in fetal development and have been implicated in intra uterine growth retardation (Hu et al. 2020; Ji et al. 2022). Studies have shown that low dose of baicalin enhanced angiogenesis by promoting cell proliferation and upregulating the expression of angiogenic genes, potentially supporting improved fetal growth and development (Zhang et al. 2011; Zhu et al. 2016). This suggests that baicalin may play a role in fetal development and contribute positively to piglet birth weight. However, the small number of replicates used in this study limits strong conclusions about the effect of SB supplementation on birth weight, but further research with larger sample sizes, ideally on commercial farms, is needed to clarify the magnitude effect of SB supplementation on birth weight.

The SB supplemented sows exhibited a proportionately, although not statistically significant, lower number of piglets weighing below 8 kg at weaning. This finding bears valuable practical implications for pig producers, given that weaning weight is an essential metric for lactation performance of the sows as well as subsequent performance of the pigs during the subsequent nursery phase (Know et al. 2025). Interestingly, weekly litter live weight was greater, along with a tendency for better litter survival rate and a greater number of litters during lactation in SB supplemented sow. This is attributed to the tendency for greater milk yield and higher amount of protein secreted in milk observed in SB supplementation sows. The observations that SB supplemented sows tended to exhibit greater milk yield and higher body weight loss compared to the CON group, despite similar feed intake, suggest that these sows might have benefited from an increased daily feed allowance. Nevertheless, the experimental design stipulated that all sows receive the same daily feed allocation throughout the experimental period, irrespective of potential differences in outcomes. In support of the present results, Olagaray et al. (2019) reported an increased milk yield in lactating dairy cows supplemented with SB. The latter authors proposed that SB enhances milk production either by increasing the availability of substrates for milk component synthesis or by modulating the metabolic pathway involved in milk components synthesis in the mammary epithelial cells. On the other hand, baicalin has been shown to reduce intracellular ROS and inflammatory cytokine levels, thereby mitigating apoptosis and enhance mammary epithelial cell prolifirations in vitro, which in turn associated with more milk yield in bovine species (Pollott 2004; Perruchot et al. 2019; Yang et al. 2023; Kong et al. 2024). However, such evidence is lacking in the lactation physiology of mammary epithelial cells in porcine species. The tendency for sows supplemented with SB to experience greater body weight loss during lactation can be partly attributed to the relatively greater milk production observed in this group compared to the CON sows. Although weight loss was not statistically different among parities, second-parity sows lost more backfat than parties three and four.

### Plasma biomarkers of oxidative status and inflammation

When the level of ROS or reactive nitrogen species exceeds the cells’ capacity for neutralization, cells can counteract the adverse effects through both enzymatic and nonenzymatic antioxidant defenses (Reyes-Camacho et al. 2020; Li et al. 2022). While low to moderate levels of ROS play a beneficial role in cellular defense against pathogens and in modulating signaling pathways (Pizzino et al. 2017), excessive ROS can lead to cellular damage by impairing biological functions (Slimen et al. 2016). Supplementation with flavonoids in lactating sows has been shown to enhance antioxidant status and improve lactation performances (Meng et al. 2018; Li et al. 2022). In the present study, plasma malondialdehyde concentration was used as a marker of oxidative stress, while the activities of GPx, catalase, and SOD were used as indictors of antioxidant status. In the present study, SB supplementation did not demonstrate beneficial effect in reducing oxidative stress or inflammation. Wang et al. (2022) reported increased SOD activity on day 1 of lactation in sows supplemented with a mixture of SB and *Lonicera japonica*. Moreover, baicalin supplementation has been shown to attenuate H_2_$O_{2}^{-}$ induced ROS production in bovine mammary epithelial cells in vitro (Perruchot et al. 2019). In contrast to previous studies (Liao et al. 2021; Li et al. 2022; Wang et al. 2022), SB supplementation in the present study did not enhance the antioxidant capacity or modulate inflammatory pathways. This outcome may reflect an absence of oxidative stress or inflammation in sows, methodological limitations in the assessment of plasma biomarkers, or an insufficient level of SB supplementation to elicit measurable effects. Rubio et al. (2019) reported low precision when using serum or plasma compared to saliva for evaluating oxidative stress and antioxidant capacity biomarkers. However, the range of detectable biomarkers in saliva remains limited. Notably, the systemic blood biomarker levels measured in the present study remained below clinical thresholds (Kaiser et al. 2018a), thus SB supplementation did not provide systemic benefit in this study. Consistent with our findings, Hervé et al. (2023) reported minimal changes in inflammatory or oxidative status biomarkers in lactating sows supplemented with *Yucca shidigera* and *Quillaja Saponaria* extracts. Liang et al. (2019) evaluated the local anti-inflammatory effects of baicalin and baicalein, where the expression of NF-kB and MAPK protein and pro-inflammatory cytokines were significantly decreased in rat gut mucosa. It can be plausible that SB exerts its anti-inflammatory effects locally in the gastrointestinal tract of sows as well, highlighting the need for further studies to evaluate gut-specific inflammation either through direct analysis of gut tissue or by measuring fecal biomarkers indicative of inflammatory processes. Baicalin is poorly absorbed from the gastrointestinal tract in its native form, thus must be hydrolyzed by microflora enzymes (bacterial-glucuronidase) into its aglycone-baicalein, which is then reconverted back to baicalin, both compounds appear in plasma at low concentrations (Zhang et al. 2013). Knowing that in vitro assays with cow mammary epithelial cells showed reduced oxidative stress and NRF2 activation at the lowest dose of baicalin (Perruchot et al. 2019), a systemic effect of Scutellaria baicalensis active compound in sows cannot be ruled out, despite the lack of supporting evidence in the present study. Nevertheless, baicalin has been demonstrated to reduce inflammation by down-regulating the production of pro-inflammatory cytokines such as interleukin-6, tumor necrosis factor-alpha, interleukin-17, and interleukin-1beta (Dinda et al. 2017). However, the underlying mechanisms of *Scutellaria baicalensis* remain poorly understood (Wu et al. 2025).

Although dietary treatment did not influence plasma biomarker concentrations, their concentrations changed greatly with days in milk. Accordingly, plasma concentration of malondialdehyde was greater on day 3 of lactation, suggesting that oxidative stress was more pronounced in early lactation compared to later stage of lactation or gestation. This finding aligns with previous studies reporting that early lactation is characterized by increased oxidative stress in sows (Papadopoulos et al. 2016; Rubio et al. 2019). However, the activity of catalase was greater from birth through lactation compared to 3 days before farrowing. In contrast, the activities of SOD and GPx peaked on the day of farrowing and day 17 of lactation, respectively, compared to other days of measurements. These observations may align with the suggestion that antioxidant enzymes differ in their kinetics and mechanisms of action (Abd El 2012).

Serum hepatic biomarkers are commonly used as an indicator of liver health (Yang et al. 2014). Although previous studies demonstrated the hepatoprotective effects of baicalein supplementation in mice (Zhou et al. 2018) and chickens (Xu et al. 2021), SB supplementation in the present study did not show significant changes in concentrations of ALA, ASA, and GGT. Similarly, He et al. (2023) did not observe a difference in concentrations of AST and ALT in sows supplemented with SB compared to non-supplemented sows. However, the elevated concentrations of creatine kinase and haptoglobin observed on day 3 of lactation in this study suggest that parturition may trigger inflammatory responses ( Kaiser et al. 2018a).

### Colostrum and milk composition

Colostrum and milk are vital for suckling piglets, providing essential nutrients for growth and immunoglobulins to fight pathogens (Le Dividich et al. 2005; Quesnel et al. 2012) and antioxidants to protect against oxidative stress (Albera and Kankofer 2009; Reyes-Camacho et al. 2020). Therefore, enhancing their production through diet can improve piglet survival and growth. Although SB supplementation tended to increase milk yield in this study, it also led to reduction in milk fat and dry matter concentrations, aligning with previous findings (Ariza-Nieto et al. 2011; Hervé et al. 2023). The reduction in the concentrations of milk fat and dry matter observed in the present study is likely due to a dilution effect caused by increased milk volume. This is because, on an absolute basis, SB supplementation numerically increased the total amounts of fat and dry matter output in milk compared to the CON sows. Interestingly, the total amount of protein output in milk was greater, while that of lactose and solid-not-fat tended to be greater in SB supplemented sows. These findings justify the enhanced litter growth performance observed in the SB supplemented sows compared to the CON sows. Hojgaard et al. (2020) reported that milk intake and milk protein content account for up to 87% of the variation in piglet weight gain. In contrast to our findings, Wang et al. (2022) and He et al. (2023) reported increased concentrations of both colostrum and milk fat, as well as dry matter, respectively, in sows fed a mixture of SB and *Loicerae Flos* during late gestation and lactation. However, the impact on yield of colostrum and milk was not reported in these studies. Gheisar et al. (2018) reported no changes in the compositions of colostrum or milk in sows supplemented with a blend of SB and *Lonicera japonica* during gestation and lactation.

## Conclusion

Supplementation with SB in sow diet during late gestation and lactation improved piglet birth weight, showed a tendency to improve milk production, and supported overall litter growth during lactation. The lack of significant effects additional physiological measurements in this study may be due to the relatively small sample size per treatment group. While these constraints should be acknowledged, the positive outcomes observed, such as improved birth weight and indications of better lactational performance, highlight the potential benefits of SB supplementation. Furthermore, the underlying physiological mechanisms, particularly those affecting the maternal metabolic profile during late gestation and lactation, warrant further study.
